# A mouse model for SARS-CoV-2 infection by exogenous delivery of hACE2 using alphavirus replicon particles

**DOI:** 10.1038/s41422-020-00405-5

**Published:** 2020-08-25

**Authors:** Ya-Nan Zhang, Xiao-Dan Li, Zhe-Rui Zhang, Hong-Qing Zhang, Na Li, Jing Liu, Jia-Qi Li, Hua-Jun Zhang, Ze-Jun Wang, Shuo Shen, Zheng-Li Shi, Hong-Ping Wei, Zhi-Ming Yuan, Han-Qing Ye, Bo Zhang

**Affiliations:** 1 Key Laboratory of Special Pathogens and Biosafety, Wuhan Institute of Virology, Center for Biosafety Mega-Science, Chinese Academy of Sciences, Wuhan, Hubei 430071 China; 2 Hunan Normal University, School of Medicine, Changsha, Hunan 410081 China; 3 Wuhan Institute of Biological Products Co. Ltd., No. 1 Huangjin Industrial Park Road, Jiangxia District, Wuhan, Hubei 420115 China

**Keywords:** Molecular biology, Translation

Dear Editor,

Since the outbreak of a novel coronavirus disease (COVID-19) in late 2019, it has spread rapidly and developed into a global pandemic. As of August 12, 2020, more than 215 countries and territories around the world have reported more than 20.5 million confirmed COVID-19 cases with over 745,693 deaths (https://www.worldometers.info/coronavirus/#countries). Such harsh conditions urged scientists across the world to gear up to develop vaccines and antiviral drugs against COVID-19, which also lead to massive requirement for experimental animals.

Severe acute respiratory syndrome coronavirus 2 (SARS-CoV-2) is the causative pathogen of COVID-19. It has been demonstrated that SARS-CoV-2 uses angiotensin converting enzyme 2 (ACE2) as cellular receptor for entry into target cells. Mouse model is the most commonly used animal model for studying human diseases. However, SARS-CoV-2 fails to invade and replicate in this traditional animal model due to the structural differences in mouse ACE2 (mACE2) compared with human ACE2 (hACE2),^1^ which has become the major hurdle for COVID-19 study. Currently, several strategies have been developed to overcome this receptor incompatibility by: (i) generating transgenic mice bearing hACE2 receptor,^2–4^ (ii) establishing adenovirus hACE2 mouse model with recombinant adenovirus expressing hACE2,^5^ and (iii) adapting the SARS-CoV-2 by serial passages in the respiratory tract of mice.^6–8^

In this study, we used an alternative strategy to generate a SARS-CoV-2-sensitive mouse model by exogenous delivery of hACE2 with Venezuelan equine encephalitis replicon particles (VEEV-VRP-hACE2) (Supplementary information, Fig. S1a). VEEV is a positive sense, single-stranded RNA virus which belongs to the genus *Alphavirus*, family *Togaviridae*. Alphavirus replicon particles (VRPs), including VEEV-VRPs, represent efficient vectors for gene delivery and have been applied to studies of vaccine development, gene therapy and cell transduction. They contain self-replicating RNA-encoding viral replicase proteins (nsP1-nsP4) and express the gene of interest in place of viral structural protein genes.^9^ By providing viral structural proteins in trans, the replicon RNA is packaged into VEEV-VRPs for in vitro and in vivo gene delivery.^10^ Due to their intrinsic biological properties, VEEV-VRPs offer several advantages with a broad range of susceptible host cells, high expression level of cytoplasmic proteins and easy manipulation of recombinant RNA molecules using cDNA clones.^10,11^

Here, Venezuelan equine encephalitis virus (VEEV) replicon expressing hACE2 with a C-terminal S-tag was packaged into VRPs using the helper RNAs encoding VEEV capsid and envelope proteins to produce VEEV-VRP-hACE2 (Supplementary information, Fig. S1). MLE-12 cells (mouse lung type II epithelial cell line) were used to evaluate the availability of VEEV-VRP-hACE2 for SARS-CoV-2-sensitive cells establishment. After confirming hACE2 expression in MLE-12 cells transduced with VEEV-VRP-hACE2 (VRP-hACE2) through indirect immunofluorescence assay (IFA) (Fig. 1a) and western blotting (Fig. 1b), MLE-12 cells were administered with VEEV-VRP-hACE2 at 12 h prior to SARS-CoV-2 (WIV04)^1^ infection. The SARS-CoV-2 NP-specific IFA-positive cells were only observed in VEEV-VRP-hACE2-transduced MLE-12 cells but not in the DMEM-treated cells (Fig. 1c). Similar results were obtained using BHK-21 cells (Fig. 1c). These results demonstrated that VEEV-VRP-hACE2 could efficiently deliver hACE2 in vitro and convert nonpermissive cells into SARS-CoV-2-permissive cells.

**Fig. 1:**
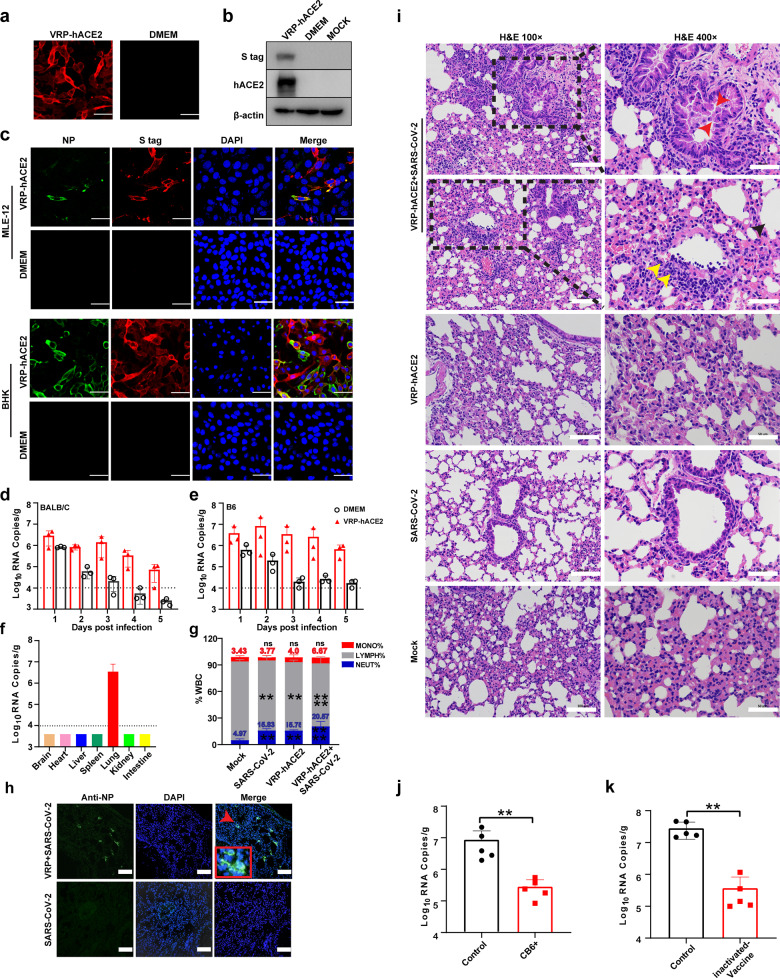
Development of mouse model for SARS-CoV-2 infection. **a**, **b** Expression of hACE2 was analyzed using IFA (**a**) and western blot assay (**b**) in MLE-12 cells transduced with VEEV-VRP-hACE2. **c** Transduction with VEEV-VRP-hACE2 converted nonpermissive MLE-12 cells and BHK-21 cells into SARS-CoV-2 permissive cells. **d**, **e** Viral RNA loads of lung tissues in VEEV-VRP-hACE2-transduced BALB/c (**d**) and C57BL/6 mice (**e**) after infection with SARS-CoV-2. **f** Viral RNA loads of different tissues in the BABL/c mice transduced with VEEV-VRP-hACE2 followed by SARS-CoV-2 infection. **g** White blood cells analysis in the peripheral blood collected from each group of BALB/c mice. **h** SARS-CoV-2 antigen detection in the lung tissues of BALB/c mice transduced with VEEV-VRP-hACE2 and infected with SARS-CoV-2 using anti-NP antibody. **i** H&E staining of lung samples from infected BALB/c mice at 4 dpi. **j** Viral RNA loads of lungs in BALB/c mice inoculated with CB6 at 2 days post challenge with SARS-CoV-2. **k** Viral RNA loads of lungs in BALB/c mice twice immunized with inactivated SARS-CoV-2 vaccines at 2 days post challenge with SARS-CoV-2. Data were expressed as means ± SD, Student’s *t* test was used to analyze the differences between two groups. **P < 0.01, ***P < 0.001. Scale bars, 50 μm (**a**, **c**), 100 μm (**h**, **i** left), 50 μm (**i** right).

We then tested whether VEEV-VRP-hACE2 could in vivo deliver hACE2 to the respiratory tract of mouse and render susceptibility to SARS-CoV-2 infection. Both BALB/c and C57BL/6 mice around 6- to 8-week-old were first administered with 10^6^ IU VEEV-VRP-hACE2 or DMEM (negative control) in a volume of 80 μL DMEM through intranasal route. After 24 h, mice from different groups were inoculated intranasally with 10^5^ PFU SARS-CoV-2, and then were monitored and weighted daily for 14 days. No obvious clinical symptoms and body weight loss were observed for mice in each group (Supplementary information, Fig. S2a). The viral replication dynamics in the lungs of mice were determined through quantification of viral genomic RNAs. As shown in Fig. 1d, e, viral RNAs remained at a steady high level (10^6^–10^7^ copies/g) in both BALB/c and C57BL/6 mice transduced with VEEV-VRP-hACE2 from day 1 to day 5 post infection and decreased to background levels (10^4^ copies/g) on day 9 in transduced BALB/c mice (Supplementary information, Fig. S2b). In contrast, the continuous decline in viral RNA levels was observed in DMEM-treated mice over the whole experimental period (Fig. 1d, e). In VEEV-VRP-hACE2-transduced mice, viral RNAs were not detected in other organs such as heart, liver, spleen, kidney, brain and small intestine except for lung (Fig. 1f). NP antigen of SARS-CoV-2 was also observed in the lungs through IFA (Fig. 1h). Similar to the results reported in SARS-CoV-2-infected transgenic mice,^3^ VEEV-VRP-hACE2-transduced, SARS-CoV-2-infected mice (VRP-hACE2+SARS-CoV-2) had a higher neutrophil-to-lymphocyte ratio in the peripheral blood at 2 days post infection (dpi) compared with the mice infected with SARS-CoV-2 (SARS-CoV-2) or transduced with VEEV-VRP-hACE2 only (VRP-hACE2) (Fig. 1g). Moreover, SARS-CoV-2 infection induced the production of viral-specific IgG antibodies in the sera of VEEV-VRP-hACE2-transduced mice, as demonstrated by IFA at 21 dpi, in contrast to the negative IFA signals observed in SARS-CoV-2 only- and VEEV-VRP-hACE2 only-treated mice (Supplementary information, Fig. S2c).

To characterize the pathological features caused by SARS-CoV-2 infection in VEEV-VRP-hACE2-transduced mice, histopathological analysis of lung sections from infected mice were performed (Fig. 1i). Hematoxylin-eosin (H&E) staining showed that following SARS-CoV-2 infection, hACE2-transduced mice developed interstitial pneumonia (Fig. 1i) characterized with large area of alveolar septal thickening, part of alveoli atrophy (black arrow), large number of inflammatory cell infiltration around blood vessels and the bronchus (yellow arrow), and a small amount of epithelia/epithelioid cells detached from the bronchus (red arrow). In contrast, only small areas of alveolar septal thickening were observed in the lungs of mock-treated mice infected with SARS-CoV-2 or mice only transduced with VEEV-VRP-hACE2. These results suggested that VEEV-VRP-hACE2-transduced mice could support SARS-CoV-2 replication and develop similar pulmonary pathology to that observed in COVID-19 patients after SARS-CoV-2 infection.

To further evaluate the availability of this mouse model for COVID-19 study, a human neutralizing antibody CB6 that is a potential agent for COVID-19^12^ and an inactivated vaccine candidate that had entered clinical trials were chosen to examine their protective effects against SARS-CoV-2 infection using this mouse model. For CB6 protective assay, viral RNAs in the lungs of VEEV-VRP-hACE2-transduced mice were quantified at 3 d after SARS-CoV-2 infection. Consistent with previous study,^12^ CB6 exhibited strong protective effects against SARS-CoV-2 infection as the viral RNAs of CB6 mAb treated group were more than twenty times lower than those of mock-treated group (Fig. 1j). Histopathological analysis of lung sections provided further evidence that in contrast to typical pulmonary features of COVID-19 lung disease displayed by mock-treated mice, no lesions were observed in antibody-treated group (Supplementary information, Fig. S2d). For vaccine protection assay, BALB/c mice were immunized twice with 5 μg inactivated vaccine through the intraperitoneal route at an interval of 14 days. The mice mock immunized with PBS were used as control. Apparent seroconversion was observed in vaccine-immunized mice at 14 days post boosting vaccination through ELISA and PRNT assays, while none of the mock-immunized mice seroconverted (Supplementary information, Fig. S2c). Following VEEV-VRP-hACE2 transduction, both groups of mice were challenged with 10^5^ PFU SARS-CoV-2 via intranasal route. The inactivated vaccine significantly protected mice from SARS-CoV-2 infection, as dramatically decreased RNA copies were detected in the vaccine-immunized mice compared with those in the mock-immunized mice (Fig. 1k).

Overall, these results show that the VEEV-VRP-hACE2-transduced mouse model can be rapidly established without any genetic manipulation (knock-out/in), and the productive viral replication and visible histopathology in transduced mice that mimic clinical COVID-19 illness allow us to test potential antiviral agents and vaccines using this model.

## Supplementary information

Supplementary information, Methods and Figures
