# Supplementary material for: A mouse model for SARS-CoV-2 infection by exogenous delivery of hACE2 using alphavirus replicon particles
Source: Cell Res. 2020 Aug 25;30(11):1046–8. doi: 10.1038/s41422-020-00405-5 (PMC7445228; doi:10.1038/s41422-020-00405-5)
Supplement: Supplementary file 1 — Supplementary information, Methods and Figures [file 41422_2020_405_MOESM1_ESM.pdf]

A mouse model for SARS-CoV-2 infection by exogenous delivery of hACE2 using alphavirus replicon particles

Ya-Nan Zhang[1]†, Xiao-Dan Li[1,2]†, Zhe-Rui Zhang[1]†, Hong-Qing Zhang[1], Na Li[1], Jing Liu[1], Jia-Qi Li[1], Hua-Jun Zhang[1], Ze-Jun Wang[3], Shuo Shen[3], Zheng-Li Shi[1], Hong-Ping Wei[1], Zhi-Ming Yuan[1], Han-Qing Ye[1]*, Bo Zhang[1]*

[1] Key Laboratory of Special Pathogens and Biosafety, Wuhan Institute of Virology, Center for Biosafety Mega-Science, Chinese Academy of Sciences, Wuhan, 430071, China;

[2] Hunan Normal University, School of Medicine, Changsha, 410081, China

[3] Wuhan Institute of Biological Products Co. Ltd., No.1 Huangjin Industrial Park Road, Jiangxia District, Wuhan, 420115, China

†These authors have equal contribution to this study

*Correspondence should be addressed to:

Han-Qing Ye, Wuhan Institute of Virology, Chinese Academy of Sciences, Wuhan 430071, China; Tel: +86-27-87197822; email: yehq@wh.iov.cn

Bo Zhang, Wuhan Institute of Virology, Chinese Academy of Sciences, Wuhan 430071, China; Tel: +86-27-87197822; email: zhangbo@wh.iov.cn

Running title: Mouse model for SARS-CoV-2 infection

Keywords: SARS-CoV-2, Angiotensin converting enzyme II, alphavirus replicon particles

Supplementary information

Materials and Methods

Ethics statement

All the mice were cared in accordance with the recommendations of National Institutes of Health Guidelines for the Care and Use of Experimental Animals. Viral infections were conducted in an animal biosafety level 3 (ABSL-3) facility at Wuhan Institute of Virology under a protocol approved by the Laboratory Animal Ethics Committee of Wuhan Institute of Virology, Chinese Academy of Sciences (Permit number: WIVA26201701).

Cells, virus and antibodies

Baby hamster kidney (BHK-21) cells and Vero-E6 cells were cultured in Dulbecco's modified Eagle's medium (DMEM) containing 10% fetal bovine serum (FBS), 100 units/mL of penicillin and 100 µg/mL of streptomycin. The mouse lung epithelial type II cells, MLE-12 were grown in RPMI 1640 medium supplemented with 15% FBS, 100 units/mL of penicillin and 100 µg/mL of streptomycin. BHK-21, Vero-E6 and MLE-12 cells were maintained in 5% CO_2 at 37 °C. SARS-CoV-2 (IVCAS 6.7512) was propagated on the Vero-E6 cells and titrated by single layer plaque assay with standard procedure. The human neutralizing antibody named CB6 of SARS-CoV-2 was a gift from Prof. Jing-Hua Yan at Institute of Microbiology, Chinese Academy of Sciences, Beijing. Rabbit anti-RP3-CoV N protein polyclonal antibody and a mouse anti-S tag monoclonal antibody for hACE2 detection were kindly provided by Prof. Bing Yan at Wuhan Institute of Virology. FITC-/HRP-conjugated goat anti-mouse/rabbit IgG, and Alexa Fluor 568-anti mouse IgG were purchased from Proteintech, China.

Plasmid construction

The infectious clone of the VEEV strain TC83, designated as pACYC-VEEV-TC83, which carries a Asc I restriction site downstream of the subgenomic promoter sequence

and a Pac I restriction site upstream of the 3'UTR ,was used to construct the replicon capable of expressing hACE2, and also the helper vectors encoding the capsid and envelop proteins. The hACE2 gene fusing an S tag sequence was amplified using an eukaryotic expression vector of hACE2 (pCAGGS-hACE2) which was provided by professor Zheng-Li Shi （Zhou et al., 2020） as template and inserted into pACYC-VEEV-TC83 at Asc I and Pac I restriction sites to replace the native structural genes of VEEV, generating the VEEV-hACE2 replicon. The plasmids containing the helper genomes encoding the capsid or envelop were constructed as described previously by Eugenia Volkova et al., （Volkova et al., Virology, 344(2): 315-27, 2006）with minor modifications. Firstly, the nucleotides of 5703-7490 nt of VEEV genome which covered most of the nsP4 gene were deleted by overlap PCR, and a TAA stop codon was added at the 3' terminus of nsP3 gene, generating VEEV-del nsP4 plasmid. The capsid and envelop sequences were then amplified using the full-length infectious clone as template, and pasted into pACYC-VEEV-TC83 at Asc I and Pac I restriction sites to construct the VEEV-del nsP4-C and VEEV-del nsP4-E helper genomes which were capable of expressing capsid and glycoproteins separately. All the plasmids were validated by DNA sequencing analysis before the subsequent experiments.

Production and titration of VRP expressing hACE2

The RNAs used for packaging VRP were *in vitro* transcribed using a mMESSAGE mMACHINE ^{TM} T7 Transcription Kit (Invitrogen) according to the manufacturer's protocols. To generate the VRP expressing hACE2, 8×10^6 BHK-21 cells in 0.8 mL ice-cold PBS were electroporated with 5 μg of each VEEV-hACE2, VEEV-del nsP4-C and VEEV-del nsP4-E RNAs in a 0.4 cm cuvette with a GenePulser apparatus (Bio-Rad) at 0.85 kV and 25 μF, pulsing three times at 3s intervals. The electroporated cells were seeded in a T75 flask and incubated at 37 °C in 5% CO_2. The supernatants of the cells were harvested at 24 h after transfection. The titer of VRP, which was expressed as infectious units (IU) per mL, was determined by IFA assay. The supernatants were serially diluted 10-fold and were used to infect BHK-21 cell monolayers in 24-well

plates. The infected cells were fixed with cold (-20 °C) 5% acetic acid in methanol, and subjected to immunofluorescence assay using the anti-S tag antibody as primary antibody.

Western blotting

BHK-21 cells were infected with VRP-hACE2 or VRP-empty at a multiplicity of infection (MOI) of 1. At 24 hpi, the cells were lysed in RIPA buffer (50 mM Tris, pH 7.4, 150 mM NaCl, 1% NP-40, 0.5% sodium deoxycholate, 0.1% SDS and supplemented with a phosphatase inhibitor cocktail). The samples were analyzed by SDS-PAGE and transferred onto PVDF membrane. The membranes were sequentially incubated with the primary antibodies against hACE2, S tag and β-actin, respectively and secondary horseradish peroxidase (HRP)-conjugated anti-mouse or rabbit IgG. The signals were detected with a chemiluminescence system (ChemiDoc; Bio-Rad) using Pierce ECL Western Blotting Substrate kit.

Immunofluorescence assay (IFA)

BHK-21 cells and MLE-12 cells were infected with VRP-hACE2 or VRP-empty at an MOI of 1 for 12 h, followed by infection with SARS-CoV-2 (MOI=1). At 24 h after SARS-CoV-2 infection, the cells were fixed with 4% paraformaldehyde and treated with 0.1% Triton-X 100 in PBS for 15 min at room temperature. For the detection of hACE2 expression, anti-S tag mouse antibody and Alexa Fluor 568-anti mouse IgG were used as primary and secondary antibodies, respectively. Viral replication was detected using anti-NP antibody and FITC-conjugated goat anti-rabbit IgG as primary and secondary antibodies, respectively. The nuclei were stained with DAPI. Fluorescence images were obtained with a Nikon confocal microscope.

Plaque assay

1×10^5 Vero-E6 cells per well were seeded into 24-well plates one day before plaque assay. A series of 1:10 dilutions were made by mixing 15 μL of virus sample with 135 μL of DMEM. Then, 100 μL of each dilution was added to individual wells of 24-well plates containing confluent Vero-E6 cells. The plates were incubated at 37℃ with 5%

CO_2 for 1 h before the layer of 2% methyl cellulose was added. After 4 days of incubation at 37°C with 5% CO_2, the cells were fixed with 3.7% formaldehyde and then stained with 1% crystal violet. Plaque morphology and numbers were recorded after washing the plates with tap water.

Transduction and infection of mice

Two kinds of mouse models were used in this study: six to eight weeks-old female BALB/c and C57BL/6 mice were provided by the Animal Centre of Wuhan Institute of Virology. For animal model establishment, mice were anaesthetized with Avertin (250 mg/kg) and intranasally infected with 1.6×10^6 FFU VRP-hACE2 in 80 μL DMEM per mouse one day before SARS-CoV-2 infection. The second day, mice intranasally infected 1×10^5 PFU SARS-CoV-2 in a total volume of 50 μL DMEM following weighed and observed for clinical signs daily throughout the study. And at the indicated times after SARS-CoV-2 infection, lungs or other organs including heart, liver, spleen, brain, kidney, eyes and small intestines were collected.

RNA extraction and Real-Time Quantitative RT-PCR.

Mouse organs were homogenized in DMEM, and viral RNA was extracted using QIAamp viral RNA mini kit（52906，Qiagen）following the manufacturer's protocol. qRT-PCR were performed using Luna® Universal Probe One-Step RT-PCR Kit (E3006). The primers and probe based on the SARS-CoV-2 S gene were designed as:

RBD-qF1: 5'- CAATGGTTTAACAGGCACAGG-3'

RBD-qR1: 5'- CTCAAGTGTCTGTGGATCACG-3'

Probe: ACAGCATCAGTAGTGTCAGCAATGTCTC

Enzyme linked immunosorbent assay (ELISA)

SARS-CoV-2 antibody titer of serum samples collected from immunized mice was determined by indirect ELISA assay. 96-well microtiter plates were coated with inactivated SARS-CoV-2 at 2-8°C overnight, and blocked with 5% skim milk for 1 h at room temperature. Diluted sera were applied to each well for 2 h at 37°C, followed by incubation with goat anti-mouse antibodies conjugated with HRP for 1 h at 37°C

after 3 times PBS wash. The plate was developed using TMB, following 1M H_2SO_4 addition to stop the reaction, and read at 450 nm by ELISA plate reader for final data.

Plaque reduction neutralization test (PRNT)

The neutralizing activities of serum samples from inactivated SARS-CoV-2 immunized mice were performed by plaque reduction neutralization test (PRNT). Briefly, approximately 50 PFU of SARS-CoV-2 was pre-incubated with 2-fold serial dilutions of heat-inactivated mouse sera (starting at 1:10 dilution) for 1 h at 37℃ and then the mixture was added to Vero-E6 cell monolayers in 24-well plates and removed after 1 h of incubation followed quantifying virus by plaque assay as described above. Neutralizing antibody titers (PRNT50) were determined to be the highest serial dilutions for which the virus plaque count was reduced by 50% compared with the control.

Histological Analysis

Lung samples from mice were fixed with 4% paraformaldehyde, embedded in paraffin followed by sagittal sections of 4-μm thickness on a microtome, and mounted on APS-coated slides. For H&E stain, sagittal sections directly stained with H&E. For detection of SARS-CoV-2 antigen in fixed lungs, indirect immunofluorescence assay (IFA) was conducted. Briefly, the slides were deparaffinized, rehydrated and experienced heat-induced antigen retrieval with EDTA (pH 8.0) in a microwave oven. Then tissues were uniformly covered with 5% BSA for incubation at room temperature for 1 h followed by further incubation with the primary antibody (anti-RP3-CoV N protein polyclonal antibody, 1:500) and then washed in PBS. After the slices were slightly dried, tissues were covered with 488s-conjugated goat-anti-rabbit IgG (Abcam, GB25301) at 1:200 dilution. After washing in PBS, slides were stained with DAPI (Beyotime) at 1:100 dilution. The image information was collected using a Pannoramic MIDI system (3DHISTECH, Budapest) and FV1200 confocal microscopy (Olympus).

Blood sampling and biochemistry analysis

Blood was collected retro-orbitally and transferred to a blood collection tube containing EDTA to prevent clotting. Cell classification was analyzed by a ProCyte Dx Hematology Analyzer (IDEXX).

Statistical Analysis

All data were analyzed using GraphPadPrism 8.0.2 software and expressed as mean ± standard deviation (SD). The statistical significance was assigned when P values were < 0.05. Student's T-test was used to analyze the differences between two groups, and significant differences between groups were determined using a two-way analysis of variance (ANOVA).

Supplementary information, Fig. S1

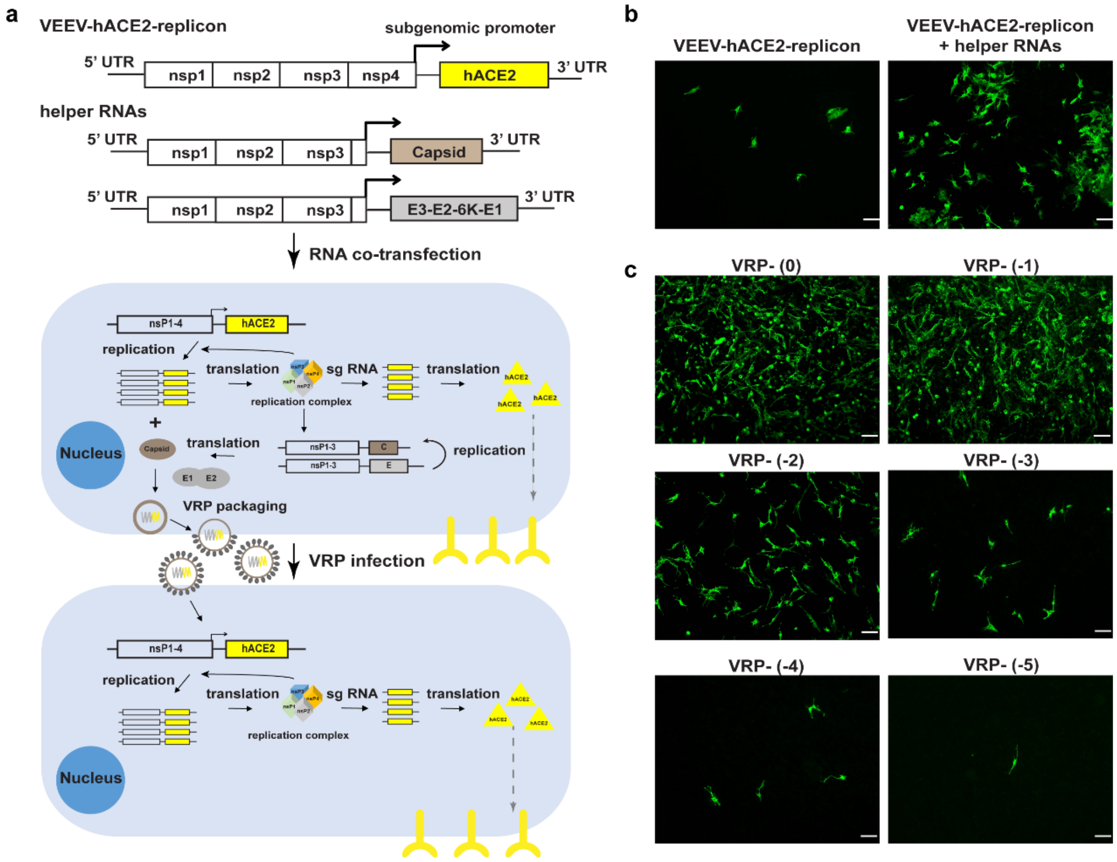

Supplementary information, Fig. S1. Packaging and titration of VEEV-VRP-hACE2. (a) Schematic representation of VEEV-hACE2 replicon and helper RNAs genomes and transduction of host cells with VEEV-VRP-hACE2. The VEEV-VRP-hACE2 packaging system include the VEEV-hACE2 replicon and two helper RNAs expressing the structural protein separately. The hACE2 gene fused with an S tag sequence was inserted to a VEEV-TC83 infectious clone to replace the native capsid-E3-E2-6K-E1 structural protein, generating the VEEV-hACE2 replicon. In the two helper RNAs, the nucleotides of 5703-7490 nt of VEEV genome covering most of the nsP4 gene were deleted, and a TAA stop codon was added at the 3' terminus of nsP3 gene, capsid and envelop gene were separately inserted and expressed. (b) To evaluate the packaging ability of the helper RNAs, BHK-21 cells were electroporated with 5 μg of VEEV-hACE2 replicon RNA, or co-electroporated with 5 μg of each VEEV-hACE2 replicon and the two helper RNAs. About 3×10^5 transfected cells were plated in 35

mm dishes, the expression of hACE2 was monitored by IFA using anti-S tag antibody at 24 hpt. The co-transfected cells generated more IFA positive cells than VEEV-hACE2-replicon transfected cells, suggesting that VRP containing VEEV-hACE2 were packaged by the capsid and envelop proteins in the co-transfected cells. (c) VEEV-VRP-hACE2 were generated by harvesting culture supernatants from BHK-21 cells co-transfected with VEEV-hACE2 replicon and the two helper RNAs at 24 hpt. To determine VRP titers, BHK-21 cells monolayer were infected with serially diluted VRPs, and the cells were fixed and subjected to IFA using anti-S tag antibody at 24 hpi. The S tag positive cells were counted to monitor the VRP titers which are represented as IU/mL. The expression level of hACE2 within cells infected with different dilution of VRP were exhibited. Scale bars are 50 μm in B and C.

Supplementary information, Fig. S2

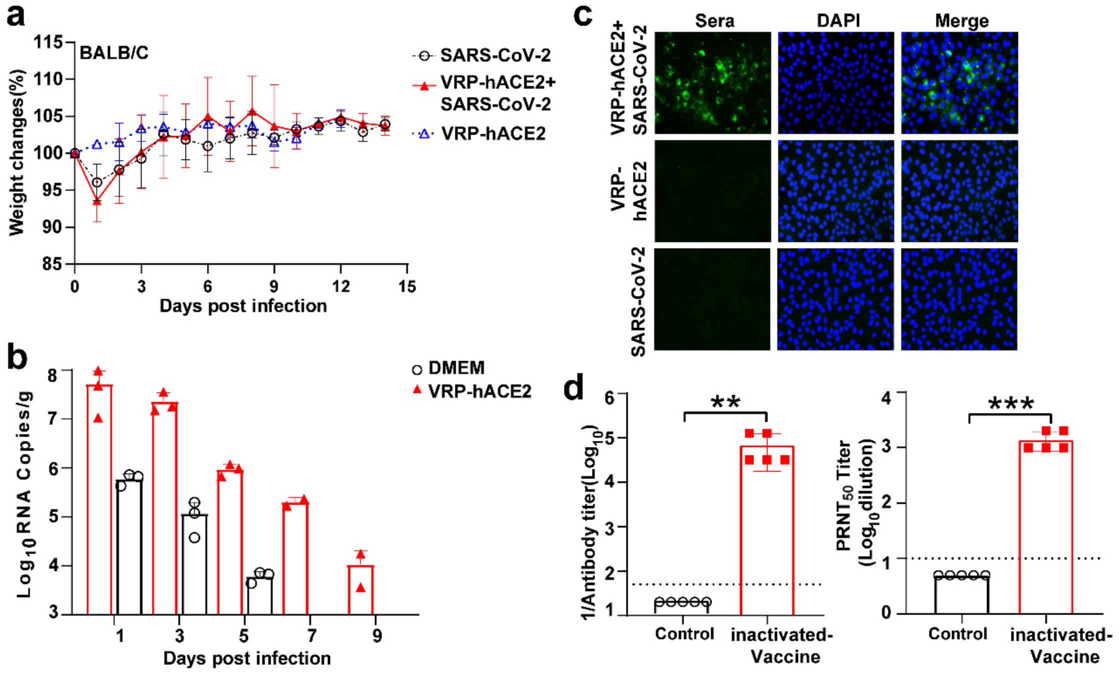

Supplementary information, Fig. S2. Development of mice model supportive of SARS-CoV-2 infection. (a) Weight changes of the VEEV-VRP-hACE2 transduced or DMEM treated BALB/c mice after SARS-CoV-2 infection during 14 days. (b) BALB/c mice were 24 h pre-administered with 1.6×10^6 IU VEEV-VRP-hACE2 or DMEM prior to infection by 10^5 PFU SARS-CoV-2. At indicated time points after SARS-CoV-2 infection, lungs of mice were collected for viral RNA level examination. Data are expressed as mean ± standard deviation (SD). (c) IFA of sera from BALB/c mice transduced with VRP-hACE2 or DMEM following infected with 1×10^5 PFU SARS-CoV-2 at 21 days. (d) Specific antibody titers of sera from mice boosted immunization with inactivated SARS-CoV-2 vaccine or PBS at 14 days (left) by ELISA assay, and neutralizing antibody titer using PRNT method (right). Data are expressed as mean ± standard deviation (SD). Statistical significance was measured using Student's T-tests. ** $P < 0.01$, *** $P < 0.001$.
